# Supporting Treatment for Anti-Retroviral Therapy (START) Together: Protocol for a pilot, randomized, couple-based intervention to promote women's ART adherence and men's engagement in HIV care in KwaZulu-Natal, South Africa

**DOI:** 10.1016/j.conctc.2022.100970

**Published:** 2022-08-18

**Authors:** Jennifer M. Belus, Alastair van Heerden, Heidi van Rooyen, Valerie D. Bradley, Jessica F. Magidson, Abigail C. Hines, Ruanne V. Barnabas

**Affiliations:** a Swiss Tropical and Public Health Institute, Department of Medicine, Allschwil, 4123, Switzerland; b University of Basel, Basel, Switzerland; c University of Maryland, Department of Psychology, College Park, MD, 20742, USA; d Human and Social Development, Human Sciences Research Council, Pietermaritzburg, South Africa; e SAMRC/WITS Developmental Pathways for Health Research Unit Department of Paediatrics, Faculty of Health Science, University of the Witwatersrand, South Africa; f The Impact Centre, Human Sciences Research Council, Durban, South Africa; g Division of Infectious Diseases, Massachusetts General Hospital, Boston, MA, USA; h Harvard Medical School, Boston, MA, USA

**Keywords:** Couples, HIV, ART adherence, Engagement in care, Behavioral intervention

## Abstract

**Background:**

South Africa currently has the greatest number of people with HIV globally. The country has not yet met its 95-95-95 goals, with different gaps in the HIV care cascade for women and men. This paper reports on a protocol to pilot test a couple-based intervention designed to improve women's antiretroviral therapy (ART) adherence and men's engagement in care in heterosexual couples living in the Vulindlela area of KwaZulu-Natal, South Africa. Study goals are two-fold: (1) assess the acceptability, feasibility, and fidelity of the experimental intervention, START Together, and (2) collect efficacy data on START Together for women's ART adherence, men's engagement in HIV care, and the couple's relationship functioning.

**Methods:**

Women (*N* = 20) who were not engaged with ART adherence (defined via self-reported ART difficulties, record of missed clinic visits, or viral non-suppression) are the target patients; male partners are not required to know or disclose their HIV status to be part of the study. Couples are randomized 1:1 to the experimental treatment (START Together) or treatment as usual (referrals to the local clinic to support ART adherence or any other HIV-related care). START Together is a 5-session intervention based in cognitive-behavioral couple therapy, which is a skill-based intervention focusing on communication and problem-solving skills, and Life Steps, a problem-solving intervention identifying barriers and solutions to medication adherence. Couples are assessed at baseline, post-treatment (8 weeks post-randomization), and follow-up (12 weeks post-randomization).

**Conclusion:**

This study will provide preliminary implementation and efficacy data on whether this novel approach has potential to improve women and men's HIV and healthcare-related needs.

## 1 Introduction

The 95-95-95 goals were developed by UNAIDS to test, treat, and maintain antiretroviral therapy (ART) adherence for 86% of people with HIV [1]. Important changes in the HIV care landscape have propelled countries forward towards meeting these goals, including widespread rollout of HIV care for pregnant and postpartum women, improved ART regimens and their access, and a focus on key populations driving the epidemic, including the targeting of men [[2], [3], [4], [5]]. Yet in many places, including in South Africa, which has the greatest number of people with HIV, there are still significant gaps in achieving the 95-95-95 goals [5].

For women living with HIV in South Africa, 94% are aware of their status, 78% are on ART, and 72% are virally suppressed. For men, these numbers are lower at every stage of the cascade: although 91% are aware of their HIV-positive status, 53% are on ART, and only 43% are virally suppressed [5]. It is clear that despite widespread advances in HIV care, targeted and more intensive interventions are still needed to engage women and men throughout the HIV care cascade in order to reach the goals set out by UNAIDS to end the epidemic.

One promising intervention approach to improve HIV outcomes in South Africa and other similar settings is the use of couple-based interventions (CBIs), where both partners in a relationship (typically romantic relationship, though other dyads are possible) attend and participate in treatment together. Theoretical frameworks that underlie CBIs for HIV or other health issues generally assume that improved health occurs in part through improvements in the dyad's functioning [[6], [7], [8]]. Crepaz and colleagues (2015) showed in a meta-analysis that CBIs were more efficacious than individual-based interventions (where only one person participated) in improving a number of HIV-related outcomes [9]. Since then, several other CBIs have also shown promising results when addressing HIV testing [10], HIV risk reduction [11], and outcomes for pregnant women with HIV and their babies [12]. A CBI focused on improving ART adherence and engagement in care for non-pregnant women has not yet been tested [13].

One of the benefits of using a CBI is that it offers the possibility that both partners can receive benefit from the intervention, even if the treatment is not targeting both partners equally. For example, a CBI in South Africa that aimed to reduce men's risky drinking and HIV risk also had benefits for female partners who participated, through decreased HIV acquisition and improved communication with their partners [11,14]. Similarly in Kenya, a CBI delivered in the home to reduce HIV risk for pregnant women improved the couple's communication, as reported by both partners, which led to increased couple efficacy and uptake of more HIV prevention behaviors [15]. Engaging men in care, which has historically been very challenging [16], may be better addressed for some men through the use of a CBI, as there is evidence that men will engage in care to support their family or a female partner [17]. Framing the intervention as one to improve women's ART adherence may be a more palatable message for men, who otherwise would not participate or prioritize their own healthcare.

### 1.1 Trial objectives

The goal of the current trial is to assess (1) feasibility, acceptability, and fidelity of delivering a CBI that is framed as improving women's ART adherence and (2) efficacy of the intervention on women's ART adherence, men's engagement in HIV care, and the couple's relationship functioning. This manuscript provides an overview of the protocol and is written in accordance with the Standard Protocol Items: Recommendations for Interventional Trials (SPIRIT) guidelines.

## 2 Methods

### 2.1 Trial design

The study uses a randomized controlled trial design with two arms: (1) the experimental intervention, START Together, and, (2) treatment as usual (TAU), which serves as the control group. Couples who are eligible for study participation are randomly assigned to either TAU or START Together at a ratio of 1:1. Randomization sequence is generated using the randomization module on REDCap, the web-based platform used for data entry [18]. The randomization sequence is not revealed until study staff click ‘randomize’ in REDCap.

### 2.2 Participants and study procedures

#### 2.2.1 Eligibility criteria

**Fig. 1:**
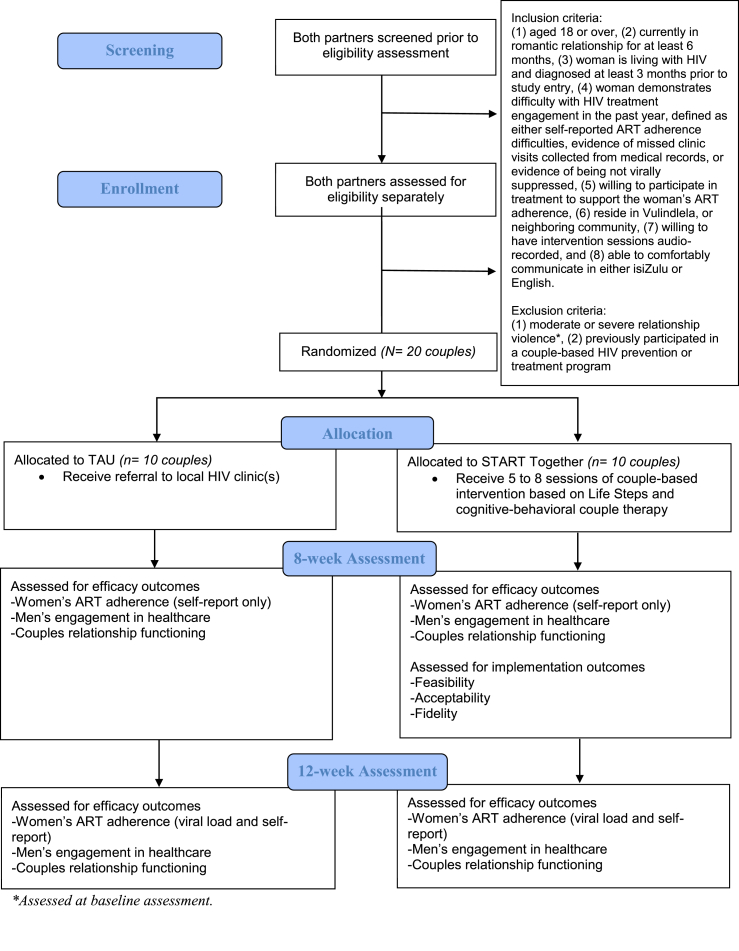
CONSORT diagram for START Together pilot trial.

The inclusion criteria for couple eligibility for the study are the following: (1) aged 18 or over, (2) currently in a committed, heterosexual, monogamous romantic relationship for at least 6 months, based on self-report, (3) woman is living with HIV and diagnosed at least 3 months prior to study entry, (4) woman demonstrates difficulty with HIV treatment engagement in the past year, defined as either self-reported ART adherence difficulties, evidence of missed clinic visits collected from medical records, or evidence of not being virally suppressed, defined as ≥ 50 copies/mL based on local standards [19], (5) willing to participate in treatment to support the woman's ART adherence, (6) reside in Vulindlela, or neighboring community, defined as spending ≥4 nights per week in the community, (7) willing to have intervention sessions audio-recorded (if randomized to START Together), and (8) able to comfortably communicate in either isiZulu or English. We chose to enroll women who demonstrate any barriers to ART adherence (either via clinic records, self-report, or viral non-suppression) given the evidence that any barriers to care are associated with increased mortality in South African women [20]. Study exclusion criteria are either of the following: (1) report of moderate or severe relationship violence, as measured by endorsing any item on the 5-item physical violence or 3-item sexual violence subscales of the WHO Intimate Partner Violence Scale [21] or (2) either partner previously participated in a CBI for HIV prevention or treatment. Fig. 1 depicts the study's flow in a CONSORT diagram.

#### 2.2.2 Recruitment

For the current study, women are considered the “index participant” meaning that they are initially identified as potentially eligible and recruited for study participation. Women with HIV who are non-adherent to their ART are recruited from local clinics. Specifically, clinic registries identify community members who have missed their monthly clinic appointments to pick up their ART and/or who are virally unsuppressed. Potentially eligible women, from these lists or who are otherwise attending these participating clinics, are informed about the study. Women provide verbal consent to complete the screening procedures. Screening takes place over the phone or in-person in private rooms. The screening assesses preliminary eligibility for the study including demographic factors (age, relationship status, etc.) and self-reported difficulties with ART adherence. To assess relationship status, we ask women whether they are in a “committed romantic relationship.”

Women who meet the preliminary eligibility criteria via the screener are asked about their partner's desire to be involved. Contact information for the study is provided to the male partner via the female participant. The male partner then contacts study staff to undergo screening procedures to assess preliminary eligibility. Once male partners complete screening and meet preliminary eligibility, the couple undergoes the consent process together. Potential participants are informed that they cannot be deemed eligible to participate until their partners are screened and both members of the couple complete the baseline assessment.

Informed consent is completed by trained staff members. Additional consent is obtained to extract data from participants' medical charts. Once consented, each member of the couple completes their baseline assessment separately. Assessments are conducted by research assistants of the same gender as the participants, if possible. During the baseline assessment, the assessor asks participants about their willingness to participate in the study as an additional strategy to ensure participants are not being coerced into study participation. Furthermore, the baseline assessment is used to further verify that the couple is in a bona fide romantic relationship given our team's past experiences recruiting couples where non-couples have feigned being a relationship in order to enroll in the study [22]. To ascertain the couple is in a bona fide romantic relationship, we ask a few informational questions to each participant regarding the partner; responses are compared after the assessment is complete. Examples of these questions include location/area of where the partner was born, employment status, and educational attainment. Similar procedures have been used in the recruitment of couples for intervention studies at this location [10].

The baseline assessment includes a battery of measures that assess previous HIV infection and treatment history, domains pertinent to relationship functioning (e.g., trust, intimacy, sexual satisfaction), and healthcare utilization. All measures have undergone a translation and back-translation process from English into isiZulu. All women (and any men who report being HIV-positive) complete a dried blood spot test to determine viral load (one of the possible ART non-adherence eligibility criteria for women) if a recent (past 30 days) viral load test is unavailable in their medical records.

Couples who meet all eligibility and none of the exclusion criteria above are eligible for study participation and randomized to either TAU or START Together by study staff initiating the randomization function in REDCap. Post-randomization, couples complete the study interventions and assessments according to those in Table 1. For START Together couples, they must initiate the first treatment session within 12 weeks of randomization. We did not a priori set an upper limit on the length of time couples were required to complete the intervention sessions by, to allow for flexibility in this pilot study.

**Table 1:**
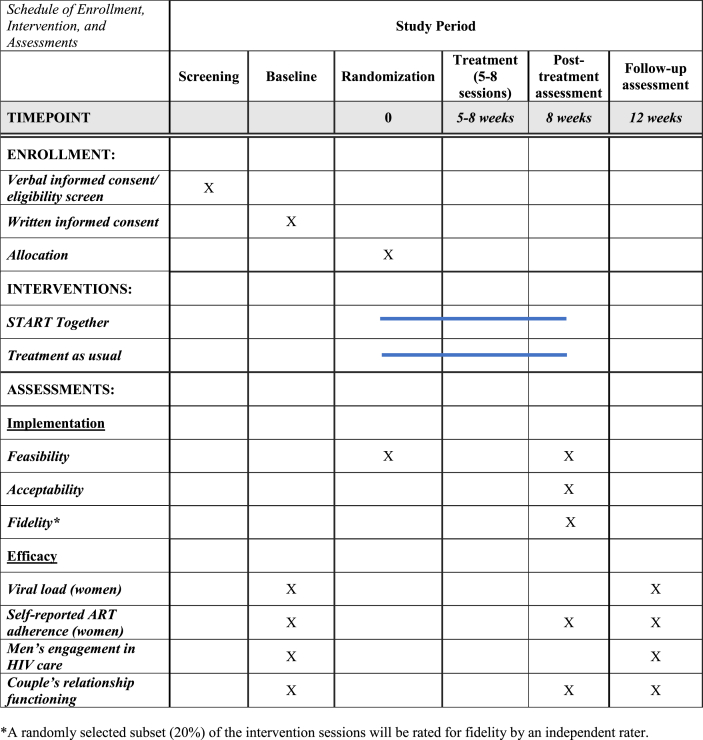
Schedule of study enrollment, intervention, and assessments.

Follow-up assessments are scheduled 8- and 12-weeks post-randomization. Each participant in the couple receives ZAR 120 cash (∼$8.50 USD) for each completed research assessment as reimbursement for their time and effort. They do not receive an incentive for attending the intervention. If couples do not complete the treatment as intended, they are still eligible to complete the research assessments. Study assessors are not blinded to couples’ treatment condition due to practical constraints of a small research team.

### 2.3 Interventions

#### 2.3.1 Treatment as usual (TAU)

Individuals in the community who need support adhering to their ART can receive adherence counseling from HIV counselors at a local clinic in the community. We elected to use TAU as our study comparator given that this is the most likely alternative for accessing care outside of the study. For the current study then, couples who are randomized to TAU receive referrals to the local clinic for ART adherence support, other HIV-related services, or additional healthcare needs.

#### 2.3.2 START Together

The 5-session intervention is based on the principles of cognitive-behavioral couple therapy (CBCT) applied to HIV treatment [7] and Life Steps for ART adherence [23]. CBCT is behavioral therapy approach focused on skills training in order for couples to improve communication and problem-solving skills, with the ultimate goal of improved relationship functioning [24]. The principles of CBCT (i.e., working with dyads, skills training related to communication and problem-solving) have been applied to the treatment of numerous medical problems including hypertension, diabetes, and chronic obstructive pulmonary disease [25]. Uthando Lwethu, a CBI with South African couples to promote couples HIV counseling and testing, used communication and problem-solving skills as central components and showed significant improvements in rates of couples testing for HIV together [10].

Formative work for the current intervention revealed the acceptability of learning and using couple-level communication and problem-solving skills in a therapeutic setting (i.e., with a counselor) by both women and men [26]. Furthermore, the intervention incorporates Life Steps, a one-session intervention based on the principles of problem-solving therapy [23]. It focuses on identifying barriers to ART adherence as well as feasible solutions in the form of devising plans and back-up plans. Life Steps has been adapted for the South African context [27] and has previously been used to promote ART adherence in individuals with depression [28] and substance use [29] in South Africa. Life Steps has previously been adapted for delivery to dyads as an optional session for PrEP adherence in Uganda [30] and in the US for serodiscordant male couples [31].

For the current study, the intervention has 5 sessions of fixed content. However, couples can complete up to 3 additional booster sessions (i.e., 8 sessions total), depending on the couple's preference or need for additional support. This is decidedly jointly with the intervention provider (i.e., interventionist). Couples who enroll in treatment for more sessions spend more time addressing barriers to ART adherence or other HIV or relationship issues. Both partners must be present for a session to be carried out—couples are made aware of this during the informed consent process. Intervention sessions take between 60 and 75 min. Table 2 presents an overview of the content for the 5-session intervention.

**Table 2:** Intervention content for START Together.

Session	Treatment content	Homework
1	- Provide psychoeducation on symptoms, etiology, and treatment for HIV - Disabuse misconceptions about HIV transmission and infection - Describe impact of HIV on couple's relationship - Elicit individual and couple-level goals for treatment - Highlight the importance of homework assignments	

2	- Teach couple communication skills (sharing thoughts and feelings technique) - Couple practices communication skills on HIV-related topic	- Practice sharing thoughts and feelings technique on topic of choice related to HIV (low to moderate emotional intensity)

3	- Homework check-in - Discuss Life Steps to identify most challenging barriers to ART adherence - Teach couple-level problem-solving skills - Couple uses couple-level problem-solving skills to address one barrier from Life Steps	- Implement plan from Life Steps to address barrier to ART adherence - Practice problem-solving skills on another Life Steps barrier or another topic of choice related to HIV (low to moderate emotional intensity)

4	- Homework check-in - Use Life Steps and couple-level problem-solving to address additional barriers to ART adherence or other issues related to HIV (e.g., maintaining a safe and fulfilling sexual/physical relationship, decision to disclose HIV status to others)	- Implement solution to problem identified in session - Practice sharing thoughts and feelings technique or problem-solving on a topic of choice related to HIV (low to moderate emotional intensity)

5	- Homework check-in - Discussion of treatment successes and relapse prevention - Couple problem-solves barriers to maintaining gains from treatment or dealing with lapses/relapses - Couple identifies and discusses areas for desired future growth (their own or dyadic) and decides whether additional treatment sessions would be helpful	- Continue practicing new learned behaviors, including communication and problem-solving skills, as well as the devised plans to address ART non-adherence or other HIV-related issues

6–8 (booster sessions)	- Continued use of communication and problem-solving skills to address issues related to ART adherence, HIV, or the couple's relationship more broadly	- As needed, depending on the issues discussed in session

### 2.4 Intervention providers

#### 2.4.1 Selection, training, and supervision

Providers for the intervention hold a Masters degree in psychology and have experience providing clinical services for mental health. Initial training in the intervention involves a 3-day training led by the intervention developer (JMB). The training is based on previous CBI trainings with regard to content, length, and format [32]. The training involves a mix of didactics and role plays. Core concepts discussed include the CBCT model, core intervention strategies (Life Steps, communication, and dyadic problem-solving), and clinical strategies to deal with challenging couples. After the initial training, interventionists complete ongoing practice with two mock cases, which is discussed in weekly supervision. One session from each interventionist is coded for content and process fidelity (e.g., appropriate handling of couple arguments). Interventionists are required to score a 75% on fidelity to begin providing treatment.

Ongoing supervision is provided weekly (1 hour) using group supervision. The interventionist provides an English audio recording or transcription of a segment of the session, which is then reviewed by the supervisor. Verbal updates on couples whose sessions are not translated are also discussed. Feedback based on fidelity to intervention session content and process is provided. Interventionist questions regarding specific couples are also addressed. A multicultural lens is used during supervision as a framework to support differing cultural backgrounds and perspectives of the interventionist, couples, and supervisor, and facilitates open discussion about these issues in supervision [33,34].

### 2.5 Measures

#### 2.5.1 Implementation outcomes

***Feasibility.*** Defined as the suitability, fit, or utility of the intervention in the current setting [35]. This is measured using (1) the percentage of couples assigned to START Together who agree to enroll in the intervention and (2) a 14-item feasibility subscale of a validated implementation science measure designed for pragmatic mental health interventions in global low-resource settings [36]. Only couples randomized to START Together complete the feasibility measure and patients and partners complete the form separately at 8-weeks.

***Acceptability.*** Defined as the tolerability or satisfaction of the intervention [35]. This is measured by (1) assessing couple's attendance and retention in the intervention and (2) a 15-item acceptability subscale of the validated implementation science measure designed for global low-resource settings [36]. Only couples randomized to START Together complete the acceptability measure and patients and partners complete the form separately at 8-weeks.

***Fidelity.*** A randomly selected subset (20%) of the intervention sessions are translated from isiZulu to English and rated for fidelity by an independent rater who is trained in the protocol. The rater assesses content fidelity specific to the intervention, adapted from fidelity for a CBCT for depression intervention in a high-resource setting [37], as well as process fidelity (e.g., verbal communication, being non-judgmental) adapted from prior research on common therapist factors in low-resource settings [38].

#### 2.5.2 Efficacy outcomes

***Women's ART adherence.*** The amount of HIV viral copies extracted from medical records (past 30 days) or dried blood spots is used as an indicator of adherence to ART. A viral load of <50 c/mL is considered virally suppressed according to South African guidelines [19]. This is supplemented with participants' self-report of adherence to ART using the 3-item Ira Wilson adherence measure [39], which has been previously used in South Africa [40,41]. Blood samples are stored in a secure area at the Human Sciences Research Council and are tested by a local laboratory service. Viral load is collected at baseline and 12-weeks and self-report ART adherence is collected at baseline, 8-weeks, and 12-weeks.

***Men's engagement in HIV care.*** Dichotomous engagement in care (yes/no) is extracted from men's clinical records (if available) and supplemented with self-report at 12-weeks. Appropriate engagement in care depends on men's HIV status and current level of engagement. For men who are HIV-negative, receiving an HIV-test after 3 months is appropriate engagement in care. For men who are living with HIV but are not taking ART, initiating ART is the next appropriate step in the HIV care cascade. For men living with HIV who take ART but are not virally suppressed, improving adherence and achieving viral suppression is the next level of care engagement. Regardless of men's initial level of care engagement, achieving the next milestone in the HIV care cascade is recorded as positive for engagement in care.

***Couple's relationship functioning.*** A 39-item self-report measure assessing factors of relationship functioning was developed based on qualitative work assessing local definitions of healthy relationships with men and women in committed relationships from Cape Town and Vulindlela communities [42,43]. The factors are active relationship building, open communication, and couple-level problem-solving.^1^ Preliminary quantitative data from this measure show adequate reliability of the constructs, with alpha values ranging from .69 to .81 [44]. Assessment occurs at baseline, 8-weeks, and 12-weeks.

## 3 Data plan and management

### 3.1 Power, sample size, and data analysis

The primary efficacy outcome, for which a power analysis can be calculated, is women's viral suppression. Only one prior study examined the efficacy of a CBI for ART adherence with serodiscordant couples in the US and found that after a 4-session intervention, 47% of the intervention group compared to 25% of the control group (who received referrals to medical provider) had at least 90% adherence to their ART [45]. Based on this, a power analysis was conducted using G*Power 7 [46] comparing the proportion of the sample reaching viral suppression between the two treatment arms.^2^ Using a two-tailed test and equal allocation of the sample between the two treatment arms, to achieve a power of .80 would require a total *N* = 148 (*n* = 74 in each arm). Because the current study is a pilot trial focused on feasibility and acceptability of the proposed intervention and collecting preliminary efficacy data on the intervention, our sample size will be *N* = 20 couples (*n* = 10 couples in each arm). A sample size of 20 represents the smallest sample size deemed appropriate for any statistical modeling [47]. The study will therefore be underpowered to detect significant differences between study arms in the efficacy outcome. We will instead focus on the direction of effects.

Descriptive statistics will be reported for implementation outcomes. For feasibility and acceptability, the proportion of the sample meeting the definitions used to define these constructs (see Measures) will be reported. Means, standard deviations, and ranges will be reported for the self-report measures of feasibility and acceptability, as well as for fidelity. We will compare these descriptives to previous behavioral couple- and family-based interventions in sub-Saharan Africa [10,48].

To analyze the efficacy outcomes, we will use an intent-to-treat analysis, where all individuals will be analyzed according to the condition to which they were randomized. Generalized linear mixed models (GLMM) will be used to compare the two treatment arms (START Together vs. TAU) over time on women's ART adherence, men's engagement in care, and couple's relationship functioning. GLMM increases power by including all available data points and is appropriate for continuous, dichotomous, and proportional variables [49]. We will compare the groups on relevant clinical and demographic factors (e.g., age) that exist between the two treatment arms. A priori, we do not plan to include clinical covariates in the analyses given the small sample size. Moreover, although we will not be able to statistically account for variability in session attendance in the START Together treatment arm (including whether or not booster sessions were attended), this information will be provided descriptively to aid in contextualizing the efficacy results.

### 3.2 Data management

Data are collected via electronic measures (tablet or smartphone) using the data management software REDCap [18]. REDCap provides a web-based application with an intuitive interface for users to enter data and have real time validation rules at the time of entry. All information entered on REDCap is de-identified in order to protect participant confidentiality and privacy. Data entered on REDCap are double-checked for accuracy by a study research assistant. Data are downloaded from REDCap and stored on a secure server at the University of Maryland. Intervention sessions are digitally audio-recorded and are stored on a secure University of Maryland drive.

### 3.3 Ethical considerations and trial management

This study is approved by the ethics board of the Human Sciences Research Council (HSRC) in South Africa (REC no. 3/19/09/18). Any protocol modifications must first be submitted and approved by HSRC's ethics board prior to implementation. Any protocol modifications that alter study design or other significant study component will be submitted to clinicaltrials.gov. There is an institutional authorization agreement between researchers at the University of Maryland and HSRC allowing HSRC to have primary ethical oversight of the study. The use of study identification numbers for each couple and participant protects participant confidentiality. Identifiable information (e.g., participant names, contact information) is stored on a password-protected document and stored at HSRC. These data are only accessible by study data staff who require this information for study duties. Any hard copy data with participant identifiable information (e.g., consent forms) are stored in a secure data room (e.g., locked cabinets, locked room with limited access) at the HSRC study site.

With regard to trial management, the study does not have a data safety and monitoring board due to the small sample size and quick rate at which data collection is expected to be completed (within 6 months). However, we do have a study management team comprised of two experts in behavioral interventions for HIV in sub-Saharan Africa. The study management team has agreed to review study progress and provide feedback approximately three months after study initiation as well as on an ad hoc basis, should any issues arise. Furthermore, to ensure the safety of all participants enrolled we will consult with the HSRC ethics board should any serious adverse events arise. The HSRC ethics board will be notified within 24 h if a serious adverse event does occur. Adverse events are categorized according to the following domains: serious/non-serious, expected/unexpected, and related/unrelated to the study intervention. We will track negative relationship events, such as relationship dissolution, as adverse events.

The primary ethical concerns related to the current investigation are protecting participant confidentiality and risk of increased tension, distress, or conflict/violence occurring between partners. First, all study staff undergo ethical training in both social and behavioral sciences and good clinical practice, and are trained in the study's standard operating procedures. Second, couples are informed during the informed consent process that if the interventionist learns of moderate or severe violence occurring during the intervention, the therapist may decide to stop treatment if they believe that it is in the best interest of the couple. Study investigators (JMB, AVH, HVR), in consultation with the HSRC ethics board, will decide whether to stop the intervention. Participant safety will be prioritized over study completion. If participants in TAU report moderate or severe violence, a referral will be provided. There are no a priori stopping rules for the study.

## 4 Discussion

In order to reach the end of the AIDS epidemic, both women and men need to move through the HIV care cascade towards viral suppression. This means that interventions need to be tailored to support movement forward, regardless of where the person is in the cascade. Given the profile of the HIV care cascade in South Africa showing that women have higher levels of HIV status awareness, ART initiation, and viral suppression than men [16], and the abundant literature demonstrating the challenges to engage men in care [[50], [51], [52]], we decided to target women as the index patient. A similar approach is used in antenatal settings. There is an increasing awareness that male partner support and involvement are important factors for a number of relevant health outcomes including prevention of mother to child transmission of HIV [53] and decreased infant mortality [54,55]. Men demonstrate their support for their female partners in numerous ways when they are involved in their antenatal care including providing emotional, instrumental, and informational support [56], all of which would be expected to be help women better adhere to their ART regimen. Thus, we will assess whether this approach can work outside of antenatal settings with regard to engaging men in care and leveraging men's support to improve women's health.

The primary expected challenges with regard to this study relate to the use of a CBI. The three issues are (1) study enrollment and treatment attendance, (2) ensuring the safety of all participants (i.e., no physical or sexual violence), and (3) variability in men's outcome data given heterogeneity of where they are along the HIV care cascade. First, recruitment and participation in treatment is often more challenging with couples than individuals because it requires the consent, meeting eligibility requirements, and participation of both members of the couple. This can take substantial resources [22]. However, a recent CBI to promote joint HIV testing at the same field site showed that over 90% of couples attended at least one treatment session and 83% attended all four sessions [10].

The second major issue is the possibility for physical or sexual violence. Although couples who report moderate to severe violence in the past year are ineligible for the study and are provided with referrals, past intervention research has shown that only a small percentage of couples actually endorse these items during the screening and assessment process [10,11], despite high rates of intimate partner violence in South Africa [57]. Thus, it is important to be attentive to the emergence of violence during the intervention phase.

Finally, given that men can be at any point along the HIV care cascade when participating in the study, and with the study's small sample size, it will likely be more challenging to ascertain the intervention's feasibility, acceptability, and preliminary efficacy for men. This is one of the limitations of a pilot study with a heterogeneous population. Particular attention will need to be paid to where in the HIV care cascade men are when evaluating their outcome data.

### 4.1 Conclusion

Overall, this study provides the first empirical test of an intervention that seeks to simultaneously address the differing needs of men and women throughout the HIV care cascade. Study strengths include testing a theoretically driven intervention, using a standard of care control group, using objective measurement (i.e., viral load, medical chart information) where possible, and collecting both implementation and preliminary efficacy outcomes. Primary study limitation is the use of a small sample size, though appropriate for a pilot study, will limit conclusions about strength of any observed effects. Nevertheless, the results of the study will provide an initial empirical basis as to whether the proposed approach provides a promising avenue for future research.

## Ethics approval and consent to participate

This study was approved by the Human Sciences Research Council (HSRC; REC 3/19/09/19; initial approval November 23, 2018; latest approval of the most recent protocol was February 3, 2022). The HSRC is the primary institutional review board (IRB) of record. The IRB at the University of Maryland, College Park has agreed to rely on the HSRC for continuing ethics oversight using an IRB/Independent Ethics Committee (IEC) IRB Authorization Agreement (IAA; approved January 7, 2019). The ethics committee in Switzerland (Ethikkomission NordWest-und Zentralschweiz) gave permission for the study oversight to rest with University of Maryland and HSRC. The current protocol version is Version 7, approved February 3, 2022.

## Consent for publication

Not applicable.

## Availability of data and materials

Data sharing is not applicable to this article as no datasets were generated or analyzed during the current study. Public access to the full protocol, de-identified participant-level dataset, and statistical code will be made available for interested parties by emailing the study PI and after necessary ethical approval is obtained (if relevant). All study key personnel will have access to the final de-identified trial dataset without restrictions.

## Author note

Jennifer Belus was at the University of Maryland when the formative work for this study was conducted. She transitioned to the Swiss Tropical and Public Health Institute during the study preparation phase for this trial and is now at the University Hospital Basel, Division of Clinical Epidemiology, Department of Clinical Research.

## Funding

This study is funded through the 10.13039/501100000024Canadian Institutes of Health Research (CIHR) (PI: Belus) and the Dean's Research Initiative at the 10.13039/100008510University of Maryland (PI: Belus). The funding source had no role in the study design, implementation, or decision to submit this manuscript for publication. Trial registration: Clinicaltrials.gov identifier NCT03809364. Trial registered on: January 09, 2019.

## Authors’ contributions

JMB conceptualized the project idea, secured funding for this project, and oversees all aspects of the project including write-up of this manuscript. RVB, AVH, HVR, and JFM provided guidance on aspects of study design and implementation. VDB contributed to writing the manuscript and all other authors (RVB, AVH, HVR, JFM, ACH) provided substantial edits. JMB co-leads data management and ACH coordinates aspects of this study under the mentorship of JMB. All authors read and approved the final manuscript.

## Declaration of competing interest

The authors declare that they have no known competing financial interests or personal relationships that could have appeared to influence the work reported in this paper.

## Footnotes

1 The original conceptualization had emotional support/display as an additional factor of healthy relationships. However, our more recent qualitative work within this community revealed this construct was better subsumed under active relationship building and recategorized as an outcome of the other relationship factors [43].

2 We assume that an adherence rate of at least 90% would result in viral suppression.
